# Supplementary material for: Chronic consumption of alcohol increases alveolar bone loss
Source: PLoS One. 2020 Aug 20;15(8):e0232731. doi: 10.1371/journal.pone.0232731 (PMC7446912; doi:10.1371/journal.pone.0232731)
Supplement: S1 File — (PDF) [file pone.0232731.s001.pdf]

ALT

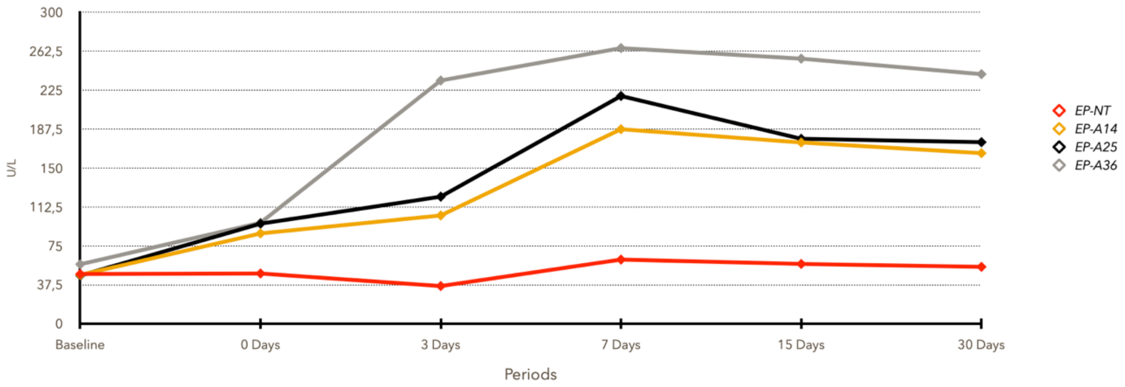

	EP-NT	EP-A14	EP-A25	EP-A36
**Baseline**	48	47,11	46,5	57,37
**0**	48,5	87,11	96,5	97,37
**3 Days**	36,4	104,4	122,5	234,4
**7 Days**	61,9	187,5	219,47	265,62
**15 Days**	57,7	174,7	178,26	255,45
**30 Days**	54,9	164,4	174,98	240,5

AST

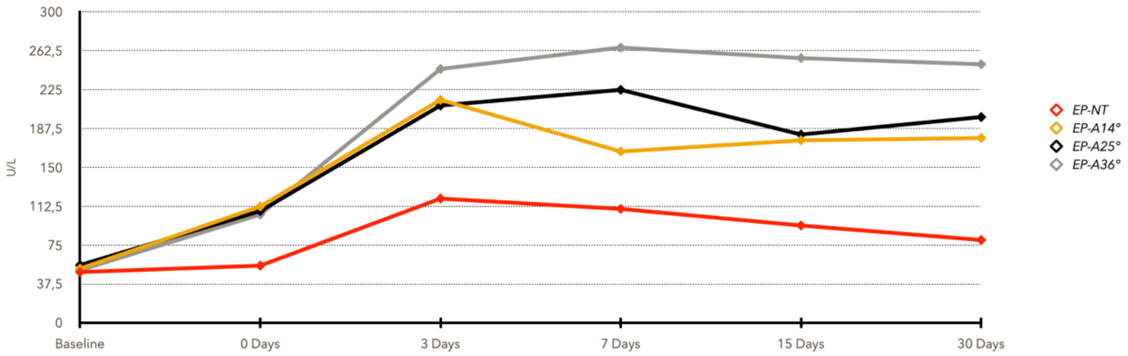

	EP-NT	EP-A14	EP-A25	EP-A36
**Baseline**	49,1	52,4	55,8	50,9
**0 Days**	55,3	112,4	108	104,62
**3 Days**	120	215,2	209,75	245
**7 Days**	110	165,37	224,87	265,62
**15 Days**	94	176,12	181,75	255,45
**30 Days**	80	178,4	198,6	249,5
