# Supplementary material for: Chronic consumption of alcohol increases alveolar bone loss
Source: PLoS One. 2020 Aug 20;15(8):e0232731. doi: 10.1371/journal.pone.0232731 (PMC7446912; doi:10.1371/journal.pone.0232731)
Supplement: S2 File — (PDF) [file pone.0232731.s002.pdf]

GROUPS

Animals	EP-NT 3 days	EP-NT 7 days	EP-NT 15 days	EP-NT 30 days	EP -A14 3 days	EP -A14 7 days	EP -A14 15 days	EP -A14 30 days	EP -A25 3 days	EP -A25 7 days	EP -A25 15 days	EP -A25 30 days	EP -A36 3 days	EP -A36 7 days	EP -A36 15 days	EP -A36 30 days
**R1**	76,99	78,94	85,34	70,08	70,09	64,23	51,34	53,36	75,75	50,39	58,37	58,78	74,68	40,80	17,73	40,85
**R2**	79,45	71,02	66,18	70,65	86,36	71,40	52,81	45,46	77,26	40,94	44,19	58,00	76,12	52,21	40,24	41,24
**R3**	79,89	66,08	75,35	83,06	75,82	45,91	38,25	46,54	74,87	65,02	59,47	71,61	77,01	51,35	30,35	30,48
**R4**	75,45	79,45	77,32	68,61	72,57	42,45	49,93	54,78	72,99	57,70	58,28	42,92	82,02	66,31	32,68	40,98
**R5**	79,98	77,40	71,09	78,03	70,04	53,11	55,11	39,18	76,97	59,37	46,15	55,21	71,77	44,67	28,24	44,13
**R6**	75,43	82,43	82,43	68,64	74,14	59,95	55,41	58,27	74,61	57,59	58,44	47,84	83,09	41,82	48,11	30,07
**R7**	75,20	87,43	68,90	74,38	79,48	64,79	54,98	41,67	69,80	49,39	51,07	37,12	77,98	52,38	44,09	26,40
**R8**	75,97	74,54	69,79	73,44	86,11	63,47	50,43	59,24	69,86	52,37	40,55	56,43	79,98	49,05	41,13	13,08
**R9**	78,76	72,45	75,43	80,87	80,90	50,24	42,78	56,53	81,20	58,50	59,66	35,43	85,98	44,05	34,35	35,86
**R10**	82,68	81,43	68,66	72,78	79,85	58,98	35,21	60,11	84,84	60,35	38,66	45,72	68,09	44,72	31,27	41,95
**MEANS**	77,98	77,12	74,05	74,05	77,54	57,45	48,62	51,51	75,81	55,16	51,48	50,91	77,67	48,74	34,82	34,50
**SD**	2,54	6,23	6,29	5,07	5,99	9,24	7,30	7,67	4,67	6,92	8,42	11,15	5,39	7,50	8,85	9,65
