# Supplementary material for: Chronic consumption of alcohol increases alveolar bone loss
Source: PLoS One. 2020 Aug 20;15(8):e0232731. doi: 10.1371/journal.pone.0232731 (PMC7446912; doi:10.1371/journal.pone.0232731)
Supplement: S3 File — (PDF) [file pone.0232731.s003.pdf]

GROUPS

Animals	EP-NT 3 days	EP-NT 7 days	EP-NT 15 days	EP-NT 30 days	EP -A14 3 days	EP -A14 7 days	EP -A14 15 days	EP -A14 30 days	EP -A25 3 days	EP -A25 7 days	EP -A25 15 days	EP -A25 30 days	EP -A36 3 days	EP -A36 7 days	EP -A36 15 days	EP -A36 30 days
**R1**	17,70	26,00	16,00	10,00	37,00	33,00	35,00	42,00	37,00	33,00	31,00	36,00	57,00	52,00	46,50	55,50
**R2**	10,40	23,00	12,00	7,70	35,00	28,00	38,00	43,50	29,00	46,00	34,60	32,00	53,00	43,00	51,50	55,50
**R3**	9,40	19,00	7,00	9,00	28,00	30,00	40,00	39,00	40,00	38,00	33,40	30,00	66,00	48,00	55,50	67,00
**R4**	10,80	23,00	6,00	6,00	20,00	31,50	30,90	38,00	28,00	34,00	29,50	41,00	56,00	53,00	51,10	59,00
**R5**	6,40	0,00	10,00	12,00	34,40	35,48	29,00	32,40	24,80	41,00	30,50	36,20	56,20	57,00	65,00	58,00
**R6**	18,00	24,00	15,00	9,70	32,00	30,48	43,00	43,00	44,00	45,60	35,00	34,00	52,40	55,00	51,00	63,00
**R7**	9,00	0,00	6,00	7,00	36,00	31,00	45,00	45,50	22,00	35,90	32,60	32,00	55,50	37,00	56,00	56,00
**R8**	8,70	24,00	9,50	11,50	25,50	31,50	32,40	37,00	34,00	43,00	32,30	28,00	65,00	54,00	48,00	57,00
**R9**	7,00	21,00	9,50	2,00	21,30	32,60	28,50	37,40	27,50	32,50	26,70	43,50	54,30	51,50	61,00	60,00
**R10**	12,00	22,00	6,00	14,50	39,60	32,40	24,00	32,00	31,30	35,00	32,40	37,70	61,00	55,50	59,00	59,00
**MEANS**	10,94	18,20	9,70	8,94	30,88	31,59	34,58	38,98	31,76	38,40	31,80	35,04	57,64	50,60	54,46	59,00
**SD**	4,00	9,77	3,67	3,50	6,81	1,99	6,82	4,56	6,94	5,15	2,48	4,84	4,77	6,27	5,91	3,64
